# The effect of metastasis location on outcome after cytoreductive surgery and heated intraperitoneal chemotherapy

**DOI:** 10.1515/pp-2022-0106

**Published:** 2022-05-23

**Authors:** Lise Hommelgaard, Jonas A. Funder, Victor J. Verwaal

**Affiliations:** Department of Surgery, Aarhus University Hospital, Aarhus N, Denmark

**Keywords:** appendix, carcinomatosis, colorectal, HIPEC, peritoneum, surgery, survival

## Abstract

**Objectives:**

This study aims to evaluate how metastases in the seven topographical regions of the simplified peritoneal cancer index (sPCI) affect the survival of patients treated with cytoreductive surgery (CRS) and heated intraperitoneal chemotherapy (HIPEC) for peritoneal carcinomatosis (PC) from colorectal (CRC) or appendiceal cancers.

**Methods:**

Data was collected retrospectively from patient records. Abdominal regions affected by PC were identified using the histological verification of surgically removed tumours found in the electronic pathology report. Verified tumours were grouped according to the sPCI topography.

**Results:**

One hundred and eighty-three patients treated with CRS and HIPEC were included. Metastases in the small bowel had a negative impact on survival with a hazard ratio of 1.89 (p=0.005). A significantly impaired survival was also detected for patients affected by metastases in the ileocolic region (p=0.01) and in the omentum and spleen (p=0.04).

**Conclusions:**

When selecting patients for CRS and HIPEC a more cautious approach may be applied by considering the regions affected.

## Introduction

The overall survival of patients diagnosed with peritoneal carcinomatosis (PC) is significant increased after the introduction of cytoreductive surgery (CRS) combined with heated intraperitoneal chemotherapy (HIPEC) [1, 2]. The procedure is currently the only possible cure to PC [3, 4] but is associated with a high risk of complications and a long period of convalescence [5]. To prevent the risk of complications without achieving reasonable benefit, proper patient selection is important. To select the right patients, efforts have been made to investigate factors affecting the outcome of the procedure and how such factors can be employed in selecting the patients benefitting from CRS and HIPEC. One such factor impacting the overall survival after CRS and HIPEC is the extent of the peritoneal involvement. This can be estimated by the Peritoneal Cancer Index (PCI) [6] or the Dutch Simplified PCI (sPCI) score [7]. High PCI-scores are linearly related to impaired overall survival and determining a PCI threshold above which the tumour burden is considered too extensive to offer CRS and HIPEC has been thoroughly investigated yet without clear results [8], [9], [10]. In 2003 Verwaal et al. found that the overall survival of patients with involvement of less than 5 of 7 regions in the sPCI-score was significantly better than that of patients with involvement of 6 or 7 regions [2]. Also, rates of post-operative complications were higher in patients with involvement of more than five regions.

Whether each of the seven regions is equally important when predicting the severity of PC has not yet been investigated. A questionnaire suggested that it is a common understanding that disease on the small bowel and on the gastro-hepatic ligament decrease chances of complete cytoreduction and thus successful treatment of the disease [11]. Our aim is to estimate the effect of metastases in each sPCI region in relation to post-operative outcome and overall survival in patients treated with CRS and HIPEC for PC from colorectal cancer and appendiceal cancer.

## Materials and methods

### Inclusion criteria

This study is based on data collected from electronic patient records of patients affected with peritoneal metastases from colorectal or appendiceal cancer. All patients were treated with CRS and HIPEC at Aarhus University Hospital, Denmark between August 1st 2015 and August 1st 2018. Aarhus University Hospital is the only facility in Denmark for HIPEC treatment. Therefore, the cohort includes all patients treated in Denmark in the given period. Patients were selected for treatment according to the Danish guidelines regarding CRS and HIPEC treatment [12]. According to these guidelines treatment was offered to patients with a histologically confirmed colorectal or appendiceal cancer with metastatic spread to the abdominal cavity. Criteria for inclusion were physiological age ≤75 years, ASA-score ≤3, WHO performance-score ≤2. Patients with less than two curable lung metastases and less than three liver metastases, of which all must be <3 cm and eligible for radio frequency ablation were eligible for treatment. Following factors were reason for exclusion from CRS and HIPEC: PCI >15–17 or >12 if curable liver metastases were present. Carcinomatosis in more than 5 of 7 sPCI regions, carcinomatosis involving the pancreatic caput and obstruction of the biliary duct were also excluding factors [12]. Obtaining completeness of cytoreduction (CCR) <2 was sought during surgery. Patients with completeness of cytoreduction (CCR) of ≥2, were not offered HIPEC and thus the present study includes primarily CCR0 and a few CCR1. A total of three included patients had CCR1. No differentiation between CCR0 and CCR1 was made.

The chemotherapeutic agent used during HIPEC procedure was either mitomycin C or oxaliplatin. Patients were included regardless of neoadjuvant chemotherapy or cancer presentation either primary or recurrent disease. The standard approach was primary surgery. All patients were discussed post-operatively at a multidisciplinary team conference where post-operative treatment regime was decided on.

### Data collection

Patient data was obtained from the electronic patient record of Central Denmark Region. It included date of birth, ASA score, duration of operation, and level of post-operative complications as graded by the Clavien Dindo classification [13]. Patients referred from outside the area covered by Aarhus University Hospital were transferred to their local hospital if no complications occurred within 7–10 days post-operatively and thus complications occurring after this period are not included if patients are transferred.

Death dates were collected from the patient records cross-referenced with the national Danish civil registration. The PCI and sPCI scores expressing the intra-operatively assessed tumour size in 13 and 7 regions respectively, were acquired from the patient record to quantify the level of abdominal involvement. Furthermore, data regarding cancer histology, status at last follow up and type of preoperative chemotherapeutic agent was obtained.

The location of metastases was extracted from the pathology report available in the patient record. The pathology report contains information from the pathological examination of the tissue resected during CRS and a histological confirmation of metastases. All pathological examinations were performed at the Institute of Pathology, Aarhus University Hospital, Denmark.

The metastases were grouped according to the topography of the sPCI which compromise seven regions: pelvis, ileocolic, greater omentum and spleen, small bowel, subhepatic andlesser omental area, right subphrenic, and left subphrenic region. The sPCI was chosen over PCI as the pathology report data was best classified according to the sPCI topography. Patients with no histological signs of metastases were grouped separately. In the pathological samples, where the topographical location was unclear, classification was not feasible.

### Statistical analysis

The end point of the present study was death of patient or date of last follow up. The survival was estimated using the Kaplan-Maier survival function. Hazard ratios were estimated using the Cox proportional hazard model and insignificant results were ruled out by the stepwise analysis. A Kruskall-Wallis test was performed to detect differences in patient characteristic within each of the seven regional groups. A log rank test was used to estimate equality in survival functions among affected and non-affected across the seven regions.

### Ethics

The study was conducted according to the guidelines of the Danish Ethical Committee. Informed consent from included patients was not required. Patients were identified from operational codes and all data were extracted from patient records and pathology reports.

## Results

A total of 233 patients underwent CRS and HIPEC at Aarhus University Hospital in the study period. A total of three patients were excluded. Two patients due to separation of CRS and HIPEC into separate procedures and one patient living abroad were excluded due to a lack of a complete patient record. Thus, data was collected from 230 patients. Forty-seven patients treated for pseudomyxoma peritonei were not included in the analysis. Following results are based on data from the remaining 183 patients. All patients underwent CRS and HIPEC as treatment for peritoneal metastases originating either from colorectal cancer (n=156) or appendiceal cancer (n=24). In three included individuals with adenocarcinoma, the origin of the primary tumour was unclear.

One hundred and five patients presented with primary cancer and 76 patients presented with recurrence of a previous cancer. Twelve patients had previously been treated with CRS and HIPEC.

All patients had histologically verified PC before the operation. Twenty-four patients had no histological signs of metastases in the per-operatively collected samples. In six patients histologically verified tumours were found but were registered as originating from locations that could not be referred to the sPCI-topography. None of the above-mentioned patients were excluded in the analysis. The median age of the patients at the time of the procedure was 64.6 years. Fifty-two patients were transferred to a regional hospital 7–10 days post-operatively.

**Figure 1:**
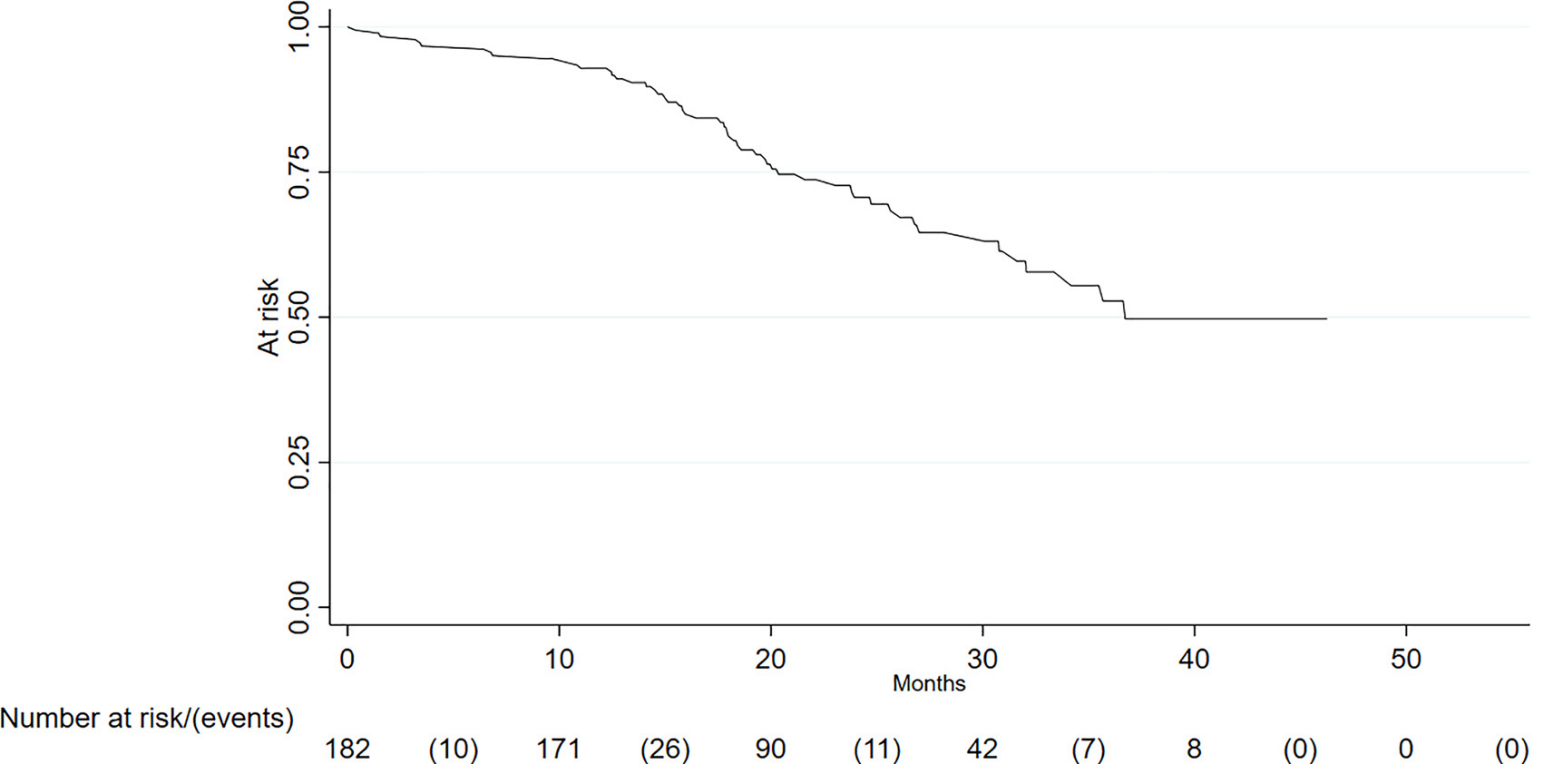
Overall survival.

The median survival after CRS and HIPEC was 36.7 months (Table 2, Figure 1) and had a range of 11 days to 46.3 months.

**Figure 2:**
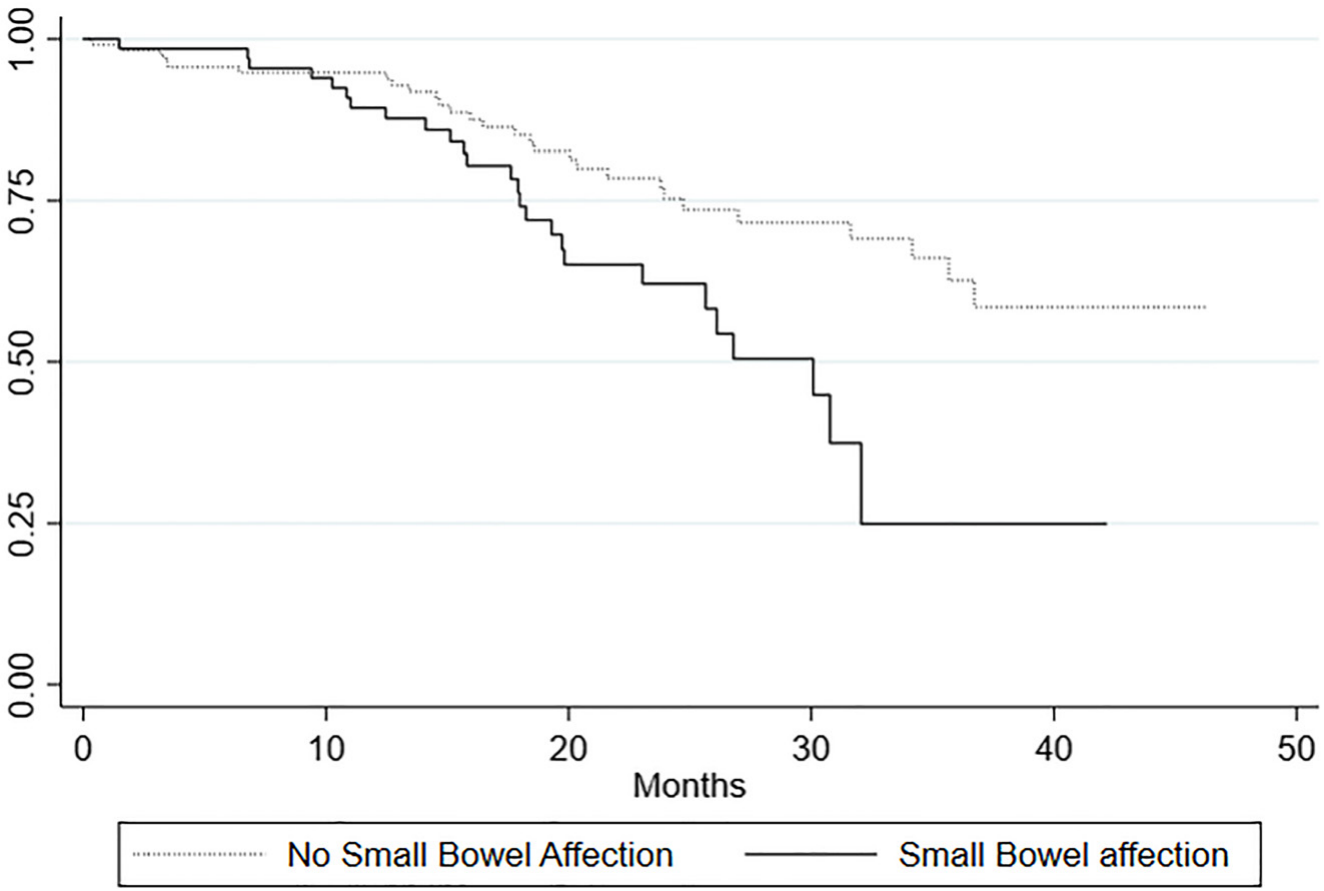
Survival by small bowel affection.

Plotting the Kaplan-Meier survival estimates of affected and non-affected patients in each group seemingly revealed an impaired survival of affected patients in all groups. We used a log rank test to test the null-hypothesis of no difference in survival between affected and non-affected only revealed significant results in three groups (Figures 2 [Fig j_pp-2022-0106_fig_003]–4). Patients with metastases in the small bowel had an impaired survival compared to the survival of the non-affected (p=0.005). In the group affected by metastases in the ileocolic region we also found an impaired survival (p=0.01) as well as for the group affected by metastases in the greater omentum/spleen area (p=0.04). The other regions showed no significant impaired survival (Table 4).

**Figure 3: j_pp-2022-0106_fig_003:**
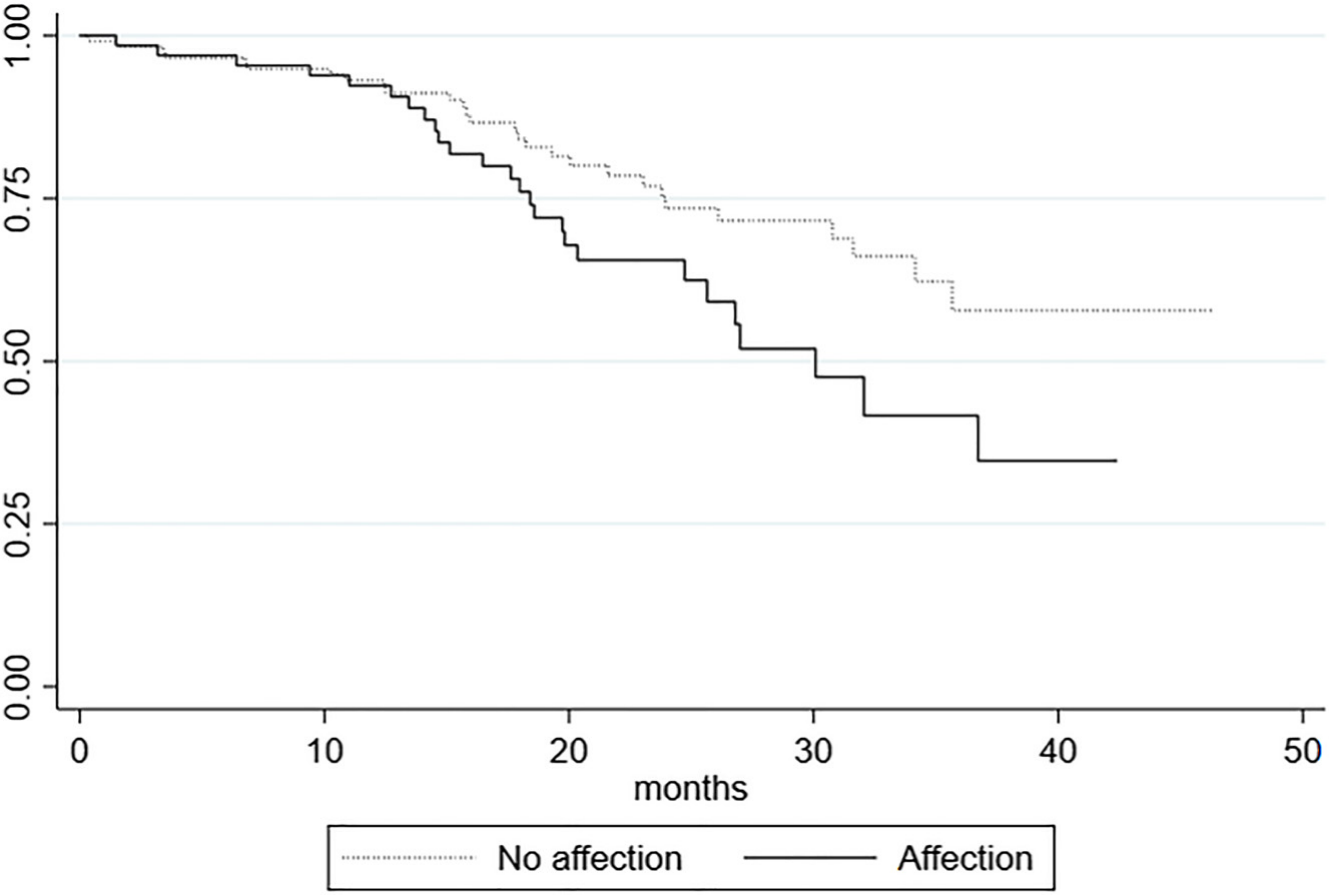
Survival by affection of omentum and spleen.

**Figure 4:**
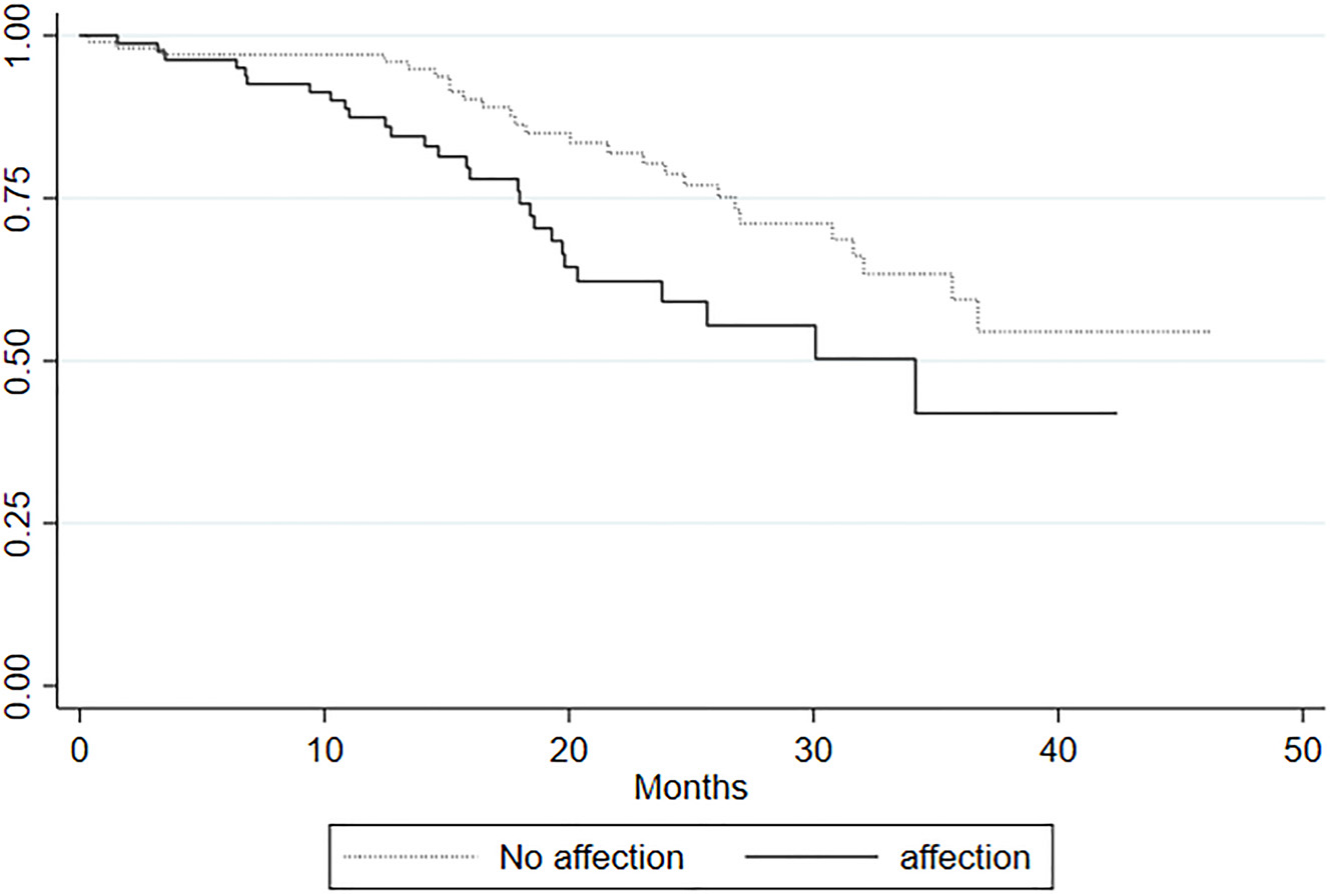
Survival by ileocolic affection.

Furthermore, a Cox regression model was performed to determine if any regions affected the survival negatively. Of the seven different regional variables only the small bowel region significantly affected the overall survival HR 1.89 [1.07–3.3], (p=0.027) after stepwise analysis was performed (Table 3).

Using the Kruskall-Wallis H-test we tested for uneven distribution of gender, cancer origin, cancer biology, ASA-score and Clavien-Dindo score in all groups. Only patients affected by metastatic spread in the pelvic regions, an uneven distribution of gender (p=0.0083), cancer origin (p=0.0008), and cancer biology (0.01) was revealed (Table 1).

**Table 1:** Patient characteristics: Total and grouped by abdominal region with histologically verified tumours.

n	Ungrouped	Grouped by histologically verified tumours						
	Total	Pelvis	Ileocolic	Omentum and spleen	Small bowel	Subhepatic	Right subphrenic	Left subphrenic
	183	106	80	65	66	15	32	8
Gender								
Male	88 (48.1%)	39	32	30	28	6	16	5
Female	95 (51.9%)	67	48	35	38	9	16	3
Age year								
Median	64.6	65.6	65.1	65.1	65.3	65.7	66.8	64.2
Cancer origin								
CRC	156	97	67	62	56	12	29	8
Appendix	24	8	10	2	8	3	2	0
Unknown	3	1	3	1	2	0	1	0
Cancer histology								
Adenocarcinoma	163	96	67	57	57	12	28	7
Signet cell	10	7	7	6	6	2	3	1
Goblet cell	10	2	3	1	1	1	0	0
ASA-score								
I	38	16	21	12	11	4	6	0
II	123	80	47	44	46	11	23	6
III	22	10	12	9	9	0	3	2
Clavien Dindo								
I	74	41	30	22	25	6	10	4
II	66	41	31	25	22	4	12	2
III	30	18	12	15	12	4	8	2
IV	11	6	6	3	7	1	1	0
V	2^a^	0	1	0	0	0	0	0

^a^Numbers do not add up as one individual died following surgery but had no histologically verified tumors.

**Table 2:** Median survival overall and for each of the seven sPCI region group.

	n	Median survival (months)	SD	95%CI
Overall	182	36.72	–	31.62–
Pelvis	106	35.67	2.2	30.08–
Ileocolic	80	34.6	4.9	20.35–
Omentum	65	30.08	3.1	24.72–
Small bowel	66	30.08	3.01	23.04–32.05
Subhepatic	15	–	–	–
Right subphrenic	32	30.08	5.06	18.34–
Left subphrenic	8	24.7	3.5	6.4–

**Table 3:** Stepwise analysis on Hazard Ratios. Independent variables.

COX stepwise	HR	SD	p	95%CI
Small bowel	1.89	0.54	0.027	[1.07–3.3]
Ileocolic	1.68	0.47	0.066	[0.96–2.9]
Right subphrenic	1.66	0.53	0.11	[0.89–3.11]

## Discussion

With the present study we evaluated how metastases in each of the seven sPCI regions affect the survival of patients with CRC or appendiceal malignancy. Patients diagnosed with pseudomyxoma peritonei were not included.

The log rank test performed on all seven groups revealed that survival is significantly impaired among patients with small bowel involvement (p=0.005), involvement of ileocolic region (p=0.01) and involvement of the omentum and spleen (p=0.04) than among patients without involvement in these regions. We did not find a significantly impaired survival among affected patients in the remaining four groups (Table 4). The median survival of patients affected by small bowel metastases was estimated to 30.1 months and to 30.1 months for patients affected by metastases in the omentum and colon which is more than 6 months shorter estimated survival time than that of the entire cohort of 36.7 months (Table 2). In the group affected by ileocolic metastases the median survival was 34.6 months. In addition to these findings the increased HR of 1.89 [1.07–3.3], which after stepwise analysis was performed was found to be significant only for small bowel patients, further add to the presumption that especially the occurrence of small bowel metastases negatively impacts the long-term survival.

**Table 4:** log rank test for survival in all seven sPCI groups.

	n	Number of deaths among affected	Median survival, months	Log rank
Total	183	54	36.72	
Pelvis	106	35	35.67	p=0.58
Ileocolic	80	28	34.6	p=0.01
Omentum & spleen	65	26	30.08	p=0.04
Small bowel	66	26	30.08	p=0.005
Subhepatic	15	4	–	p=0.96
Right subphrenic	32	13	30.08	p=0.1
Left subphrenic	8	4	24.7	p=0.27

Analysing the frequency of post-operative complications indicated no increased complication risk for any separate group. The Kruskall-Wallis H-test demonstrated uneven distributions of gender, cancer origin, and cancer histology for the pelvic region. A significantly higher frequency of female individuals (63%) in the pelvic group might be explained by the fact that metastases located in or on the surface of female internal genitalia such as uterus, ovaries and adnexae. That rectal cancer constitutes 1/3 of all colorectal cancers in Denmark [14] might explain a more frequent presentation of colorectal origin of metastases in the pelvis and thus the skewed presentation in our results.

Traditionally, studies evaluating metastatic spread in the abdominal cavity use either diagnostic imaging or the PCI/sPCI to estimate the extent of PC and tumour mass. The PCI and sPCI based on the surgeon’s visual and tactile evaluation may as well as diagnostic imaging include processes of non-cancerous origin e.g., scar tissue [15, 16]. This factor combined with an expected dispersion in the PCI-evaluation from surgeon to surgeon and institution to institution, may lead to a more inaccurate estimation of correlation between PCI/sPCI and post-operative survival and complications. Including non-cancerous processes in the survival analysis is evaded by including only histologically confirmed tumour mass in the dataset.

Data was collected from a single institution and contain information from all patients who underwent CRS and HIPEC in Denmark in the given time frame. This ensures a great level of consistency as well as a representative study cohort. All data was stored in the same electronic patient record system and all histological examinations were performed in the same institution. We experienced only limited loss to follow up on the studied parameters.

Employing the pathology reports available in the patient records enabled us to assess the post-operative histologically confirmed tumour mass and, therefore, our evaluation of metastatic spread only include actual tumours. The fact that only metastases in the small bowel, ileocolic region and in the omentum and colon yield significant findings may be a result of the categorization of samples during surgery. The three abovementioned regions constitute exact anatomical structures, whereas the boundaries of e.g., the right or left subphrenic region might be more diffuse. Furthermore, the labelling of samples in the pathology report can be difficult to locale within the sPCI topography as some samples contain structures which cannot be localized to a specific region e.g. “abdominal wall” and “umbilicus”. Thus, not all samples containing pathological tissue can be circumscribed precisely after the sPCI topography. This may result in our registration of tumour being skewed, and small bowel and ileocolic metastases and metastases in the omentum and spleen may be over-represented.

The estimated HR of 1.89 [1.07–3.3] among small bowel patients may be attributed to the fact that small bowel involvement is often associated with incomplete cytoreduction and thus less successful surgical removal of the disease [17]. This pattern may also be explained by a more aggressive nature of the colorectal and appendiceal cancers exhibiting metastatic spread to the small bowel.

The lack of significant results in especially the subhepatic and left subphrenic regions may be a result of a relatively small group of individuals being affected by metastases in these areas.

The purpose of this study was to establish whether metastatic spread to certain regions in the abdomen has a negative impact on the survival and to evaluate whether involvement of those regions can be a factor to consider when selecting patients for CRS and HIPEC treatment. The negative effects found here may lead to more careful considerations in the process of selecting patients. Taking tumour location into account when selection patients eligible for CRS and HIPEC may contribute to a decrease in post-operative morbidity and mortality.

To further investigate the results demonstrated here a more standardized way of registering the areas resected during CRS would be needed to localize the metastases of the abdominal cavity more precisely. This could simply be done by adding the number of the given sPCI region the sample was collected from to every sample collected and registered during CRS. Also, to better estimate the post-operative histologically verified tumour mass the registration of resections could include the tumour dimensions to compare our model more directly with the PCI or sPCI determined intra-operatively. A survival analysis discriminating single metastases from multiple metastases in each region could also be useful in the attempt to improve selection of patients benefitting from HIPEC-treatment.

## Conclusions

The results of our study show that patients with metastases in the small bowel have a disadvantage regarding overall survival and that HIPEC and CRS seem less beneficial for those affected. In the future these results may be employed when the operability of PC patients is considered.
